# Supplementary figures and images for: Lessons from movement ecology for the return to work: Modeling contacts and the spread of COVID-19
Source: PLoS One. 2021 Jan 22;16(1):e0242955. doi: 10.1371/journal.pone.0242955 (PMC7822505; doi:10.1371/journal.pone.0242955)

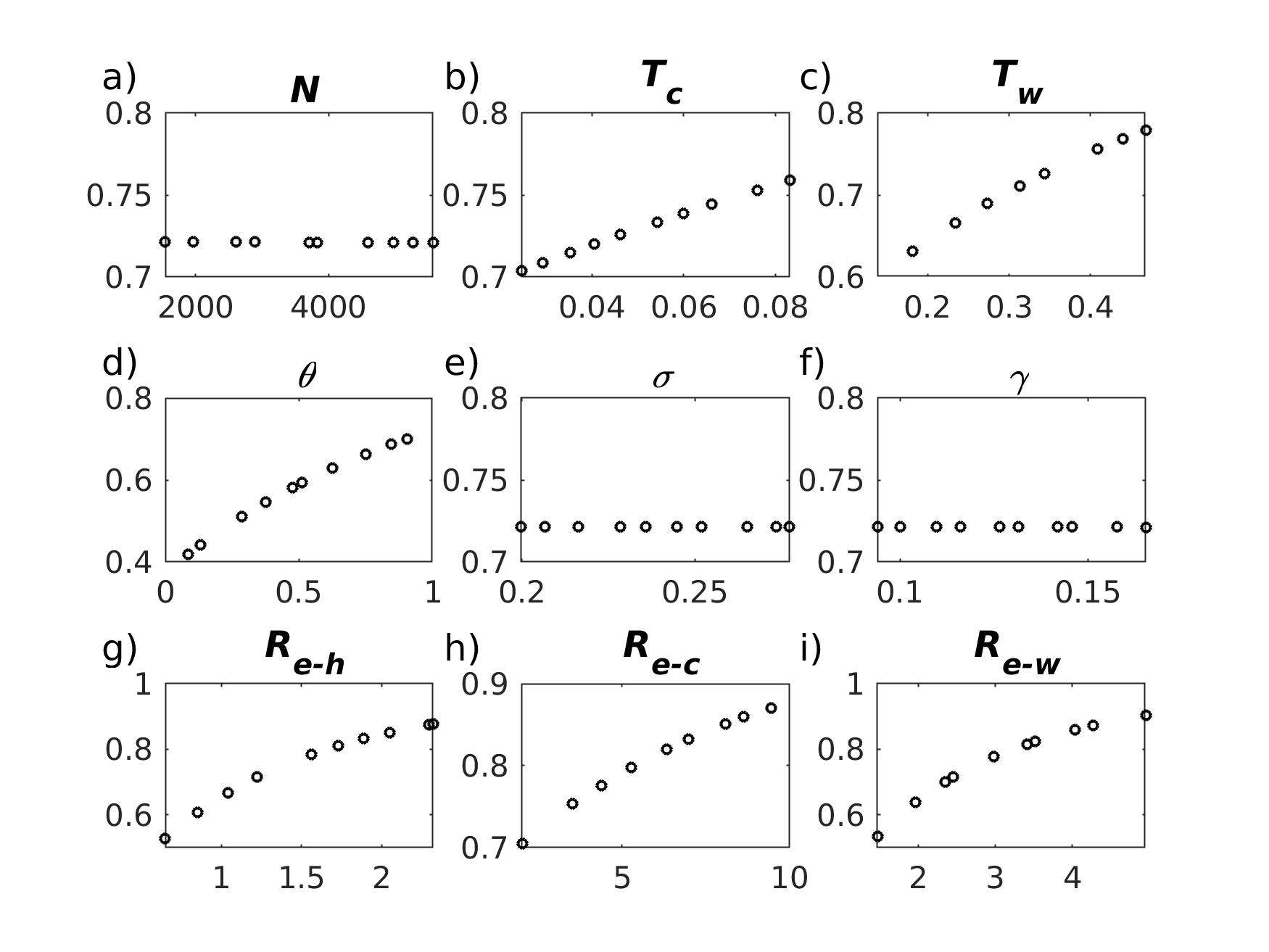

Supplement: S1 Fig — The relationship between each of the nine model parameters (x-axis) and the model output, final epidemic size (y-axis) for (a) population size (N); (b) fraction of a 24-hour day spent commuting each way for those that commute to campus (Tc); (c) fraction of a 24-hour day spent on campus for those commuting (Tw); (d) fraction of the campus population commuting to work on campus (θ); (e) recovery rate (γ); (f) effective reproductive number while at home (Re-h); (g) effective reproductive number while commuting between work and campus (Re-c); and (h) effective reproductive number while at work on campus (Re-w). (TIF) [file pone.0242955.s001.tif]

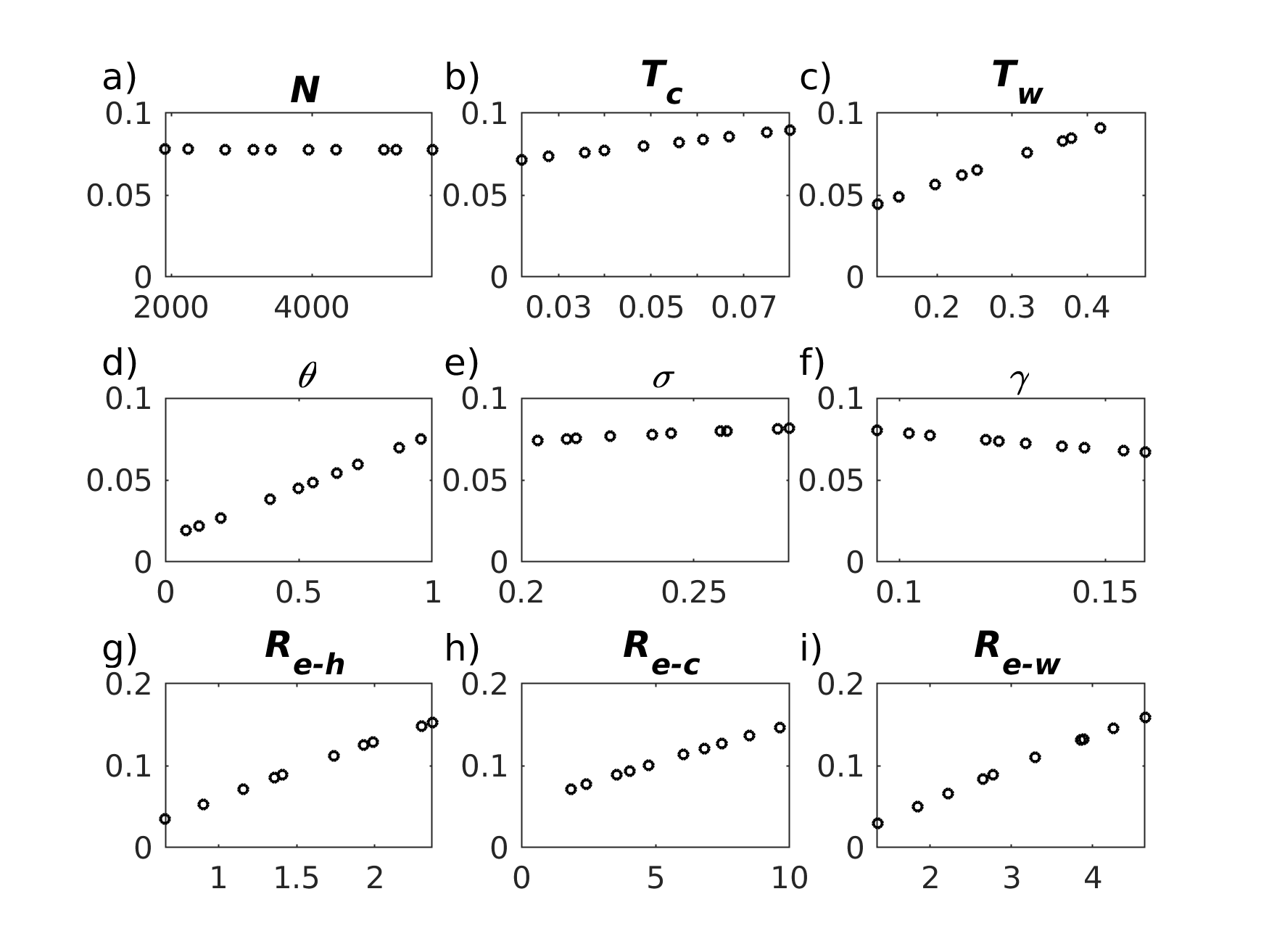

Supplement: S2 Fig — The relationship between each of the nine model parameters (x-axis) and the model output, epidemic peak size (y-axis) for (a) population size (N); (b) fraction of a 24-hour day spent commuting each way for those that commute to campus (Tc); (c) fraction of a 24-hour day spent on campus for those commuting (Tw); (d) fraction of the campus population commuting to work on campus (β); (e) recovery rate (γ); (F) effective reproductive number while at home (Re-h); (g) effective reproductive number while commuting between work and campus (Re-c); and (h) effective reproductive number while at work on campus (Re-w). (TIF) [file pone.0242955.s002.tif]

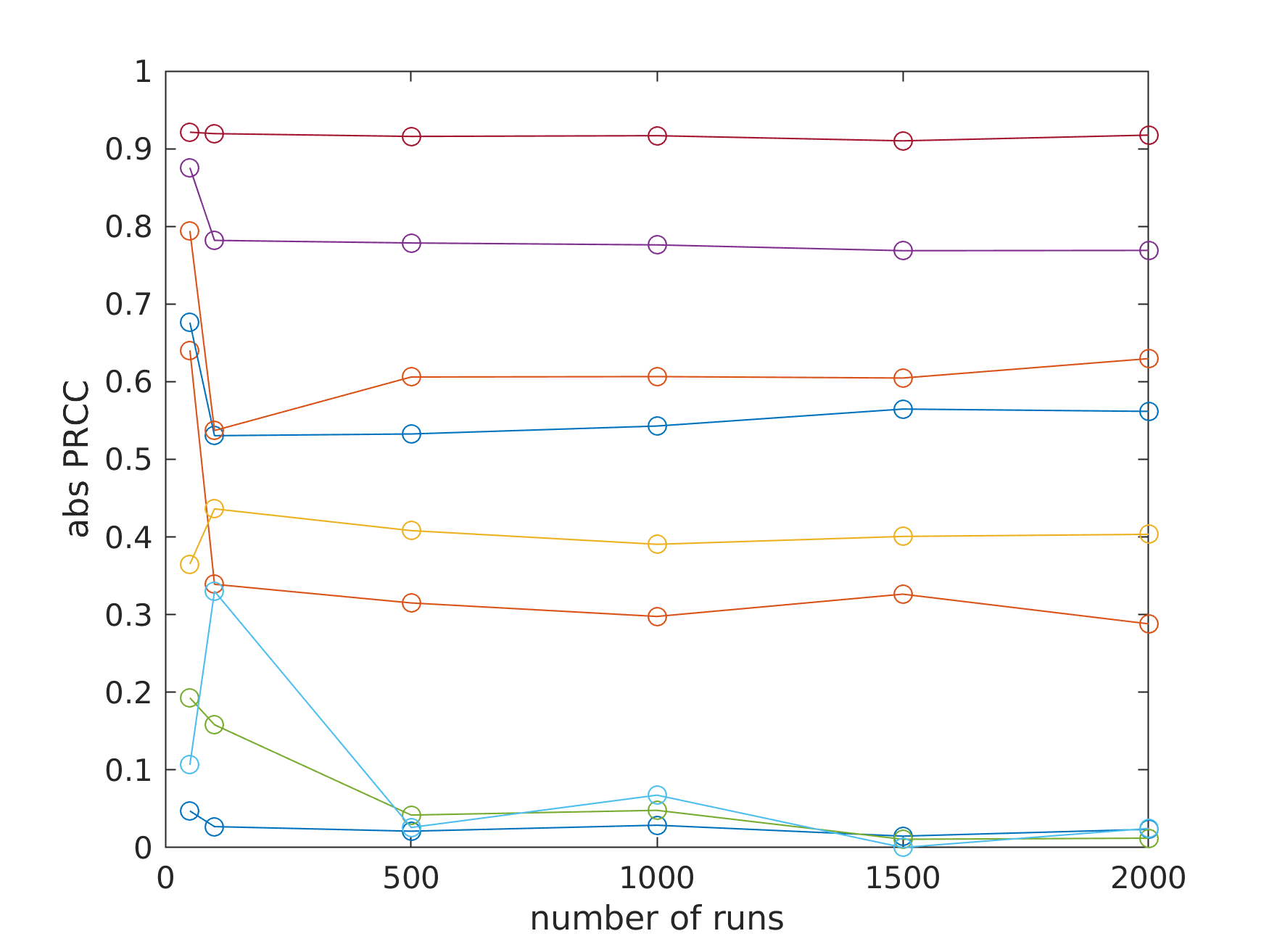

Supplement: S3 Fig — Absolute value of PRCC for the final epidemic size model output and each of the nine model parameters (N, Tc, Tw, β, σ, γ, Re-h, Re-c, Re-w) as a function of different numbers of LHS samples generated. The results seem to stabilize after about 1000 samples. (TIF) [file pone.0242955.s003.tif]

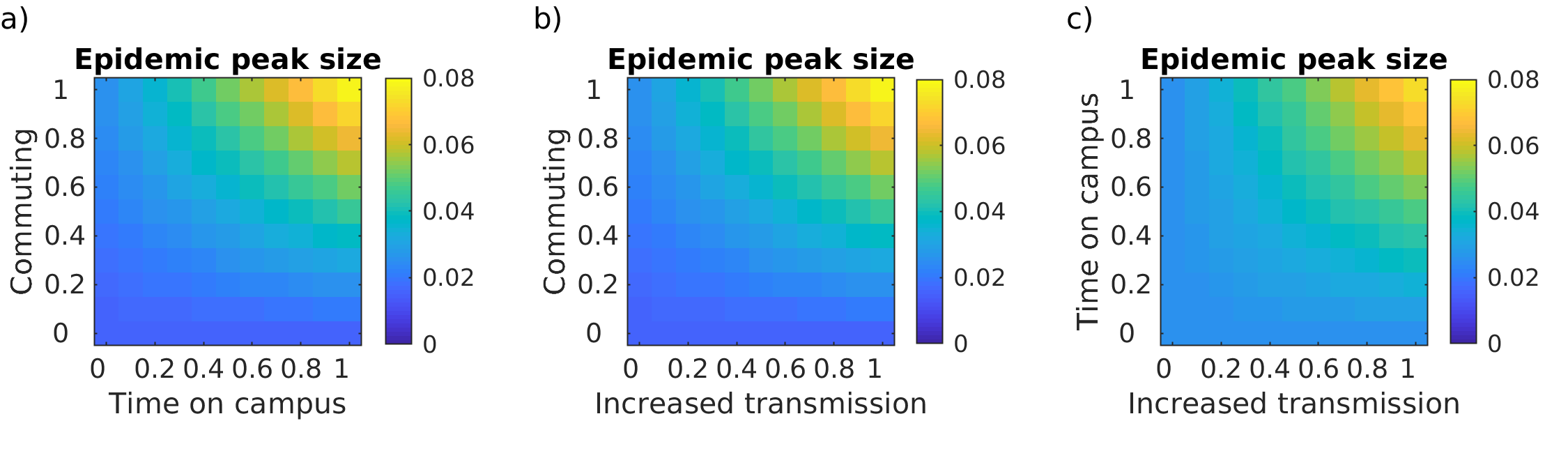

Supplement: S4 Fig — The epidemic peak size (maximum fraction of the population infected) as a function of (a) the fraction of an 8-hour workday spent on campus (x-axis) and the fraction of the population working on campus (y-axis) with no physical distancing, (b) the fraction increase in transmission while at work compared to at home (x-axis) and the fraction of the population working on campus (y-axis) with an 8-hour work day, (c) the fraction increase in transmission while at work compared to at home (x-axis) and the fraction of an 8-hour workday spent on campus (y-axis) with 100% of people on campus. (TIF) [file pone.0242955.s004.tif]

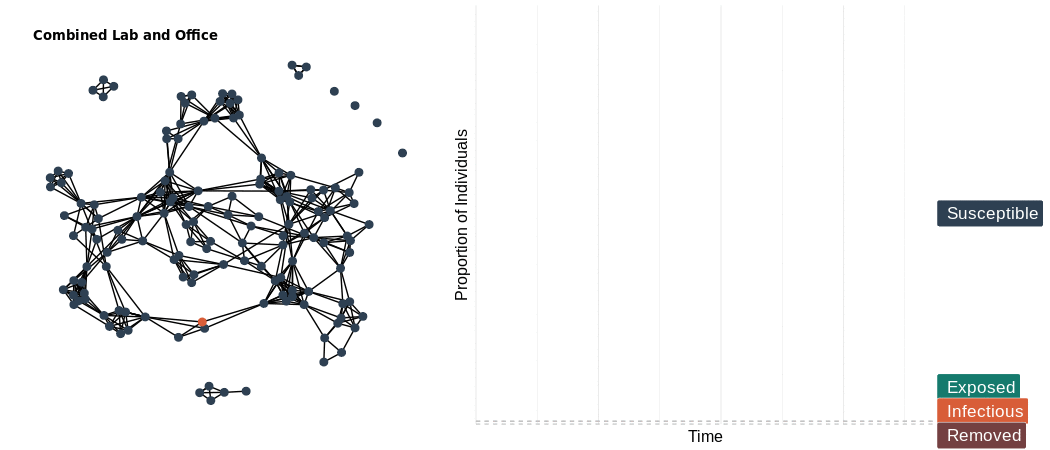

Supplement: S5 Fig — Simulations of pathogen spread across networks based on use of both shared office and lab space. (GIF) [file pone.0242955.s005.gif]

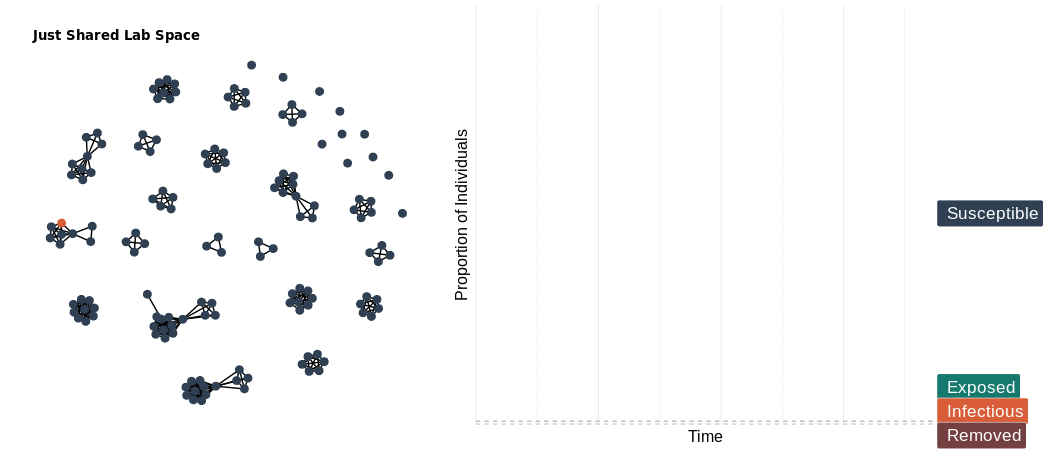

Supplement: S6 Fig — Simulations of pathogen spread across networks based on use of only shared lab space. (GIF) [file pone.0242955.s006.gif]

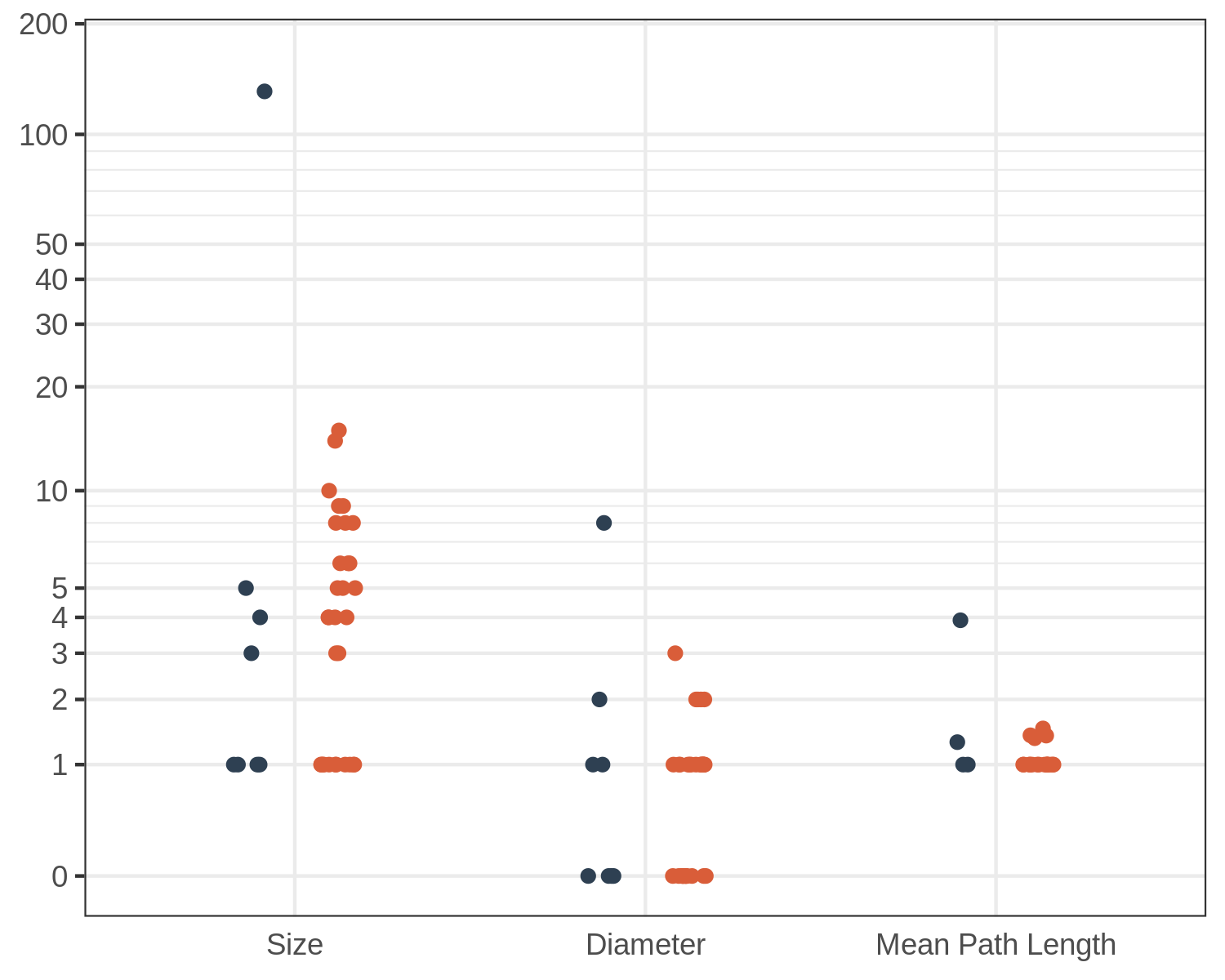

Supplement: S7 Fig — Measures of the size (number of individuals), diameter (longest shortest path between two individuals), and mean path length (average shortest path length between individuals) for each distinct component of networks presented in Fig 5A and 5B. The combined lab and office network (blue points) has 8 distinct components (8 points for each metric), while the shared lab space network contains 31 distinct components (31 points for each metric). (TIF) [file pone.0242955.s007.tif]
