# Supplementary material for: Lessons from movement ecology for the return to work: Modeling contacts and the spread of COVID-19
Source: PLoS One. 2021 Jan 22;16(1):e0242955. doi: 10.1371/journal.pone.0242955 (PMC7822505; doi:10.1371/journal.pone.0242955)
Supplement: S1 File — (PDF) [file pone.0242955.s008.pdf]

Supporting Information for:
Lessons from movement ecology for the return to work: modeling contacts and the spread of COVID-19

Allison K Shaw, Lauren A White, Matthew Michalska-Smith, Elizabeth T Borer, Meggan E Craft, Eric W Seabloom, Emilie Snell-Rood, & Michael Travisano

September 2, 2020

To evaluate the sensitivity of the network model results in the main text, here we perform further disease simulations on random graphs that are structurally similar to the empirical networks. First, we performed configuration model randomizations of each of the empirical networks. A configuration model preserves the exact degree distribution of the network, while allowing other structures to vary freely. This approach allows for the generation of "similar" networks upon which we can simulate disease dynamics and compare outcomes to those seen in the empirical networks. This model provided a fairly good fit to the combined lab and office-space network used in the main text, but fit the shared lab-space network poorly due to its highly fragmented structure. Thus, we additionally generated random graphs that kept the approximate number and size of isolated components as the shared lab-space network.

We provide additional details and figures for each of these cases below. Code to replicate these analyses can be found at `https://github.com/whit1951/EEBCovid`.

Configuration model

To generate the random graphs with degree sequences matching those of the empirical networks, we utilized the `sample_degseq` function in the `igraph` [Csardi and Nepusz, 2006] package for `R` (Version 4.0.2; R Core Team, 2020). We generated 200 random graphs for each of the two empirical networks from the main text. For each random graph, we simulated 50 epidemics as detailed in the main text, recording the epidemic peak (the maximum number of concurrently infectious individuals), the final epidemic size (the total number of individuals infected over the course of the epidemic), and the time to epidemic peak (the number of days before the epidemic peak is reached) for each simulation.

For the combined lab- and office-space network, this produced networks with similar, but in general less fragmented structure (figs. S8 and S9), which yielded similar, though slightly larger, disease dynamics (*i.e.* higher peak, more total infected, and longer times to epidemic peak; fig. S10). This discrepancy is likely a consequence of the reduced fragmentation in randomized networks compared to the empirical networks.

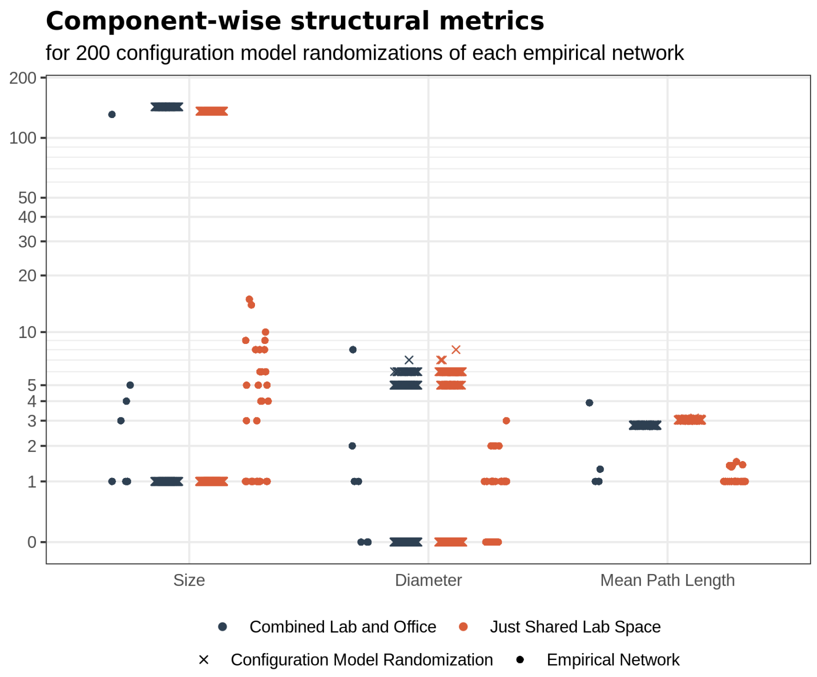

Figure S8: As fig. S7 in the supporting information, but including results from configuration model randomizations of the two empirical networks.

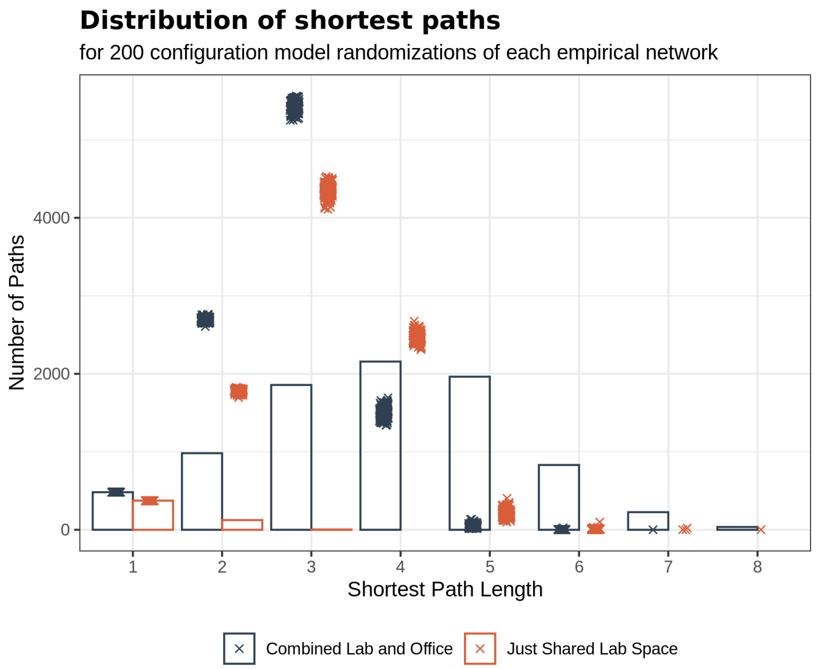

Figure S9: As fig. 5 C in the main text (histogram), but including results from configuration model randomizations of the two empirical networks (points).

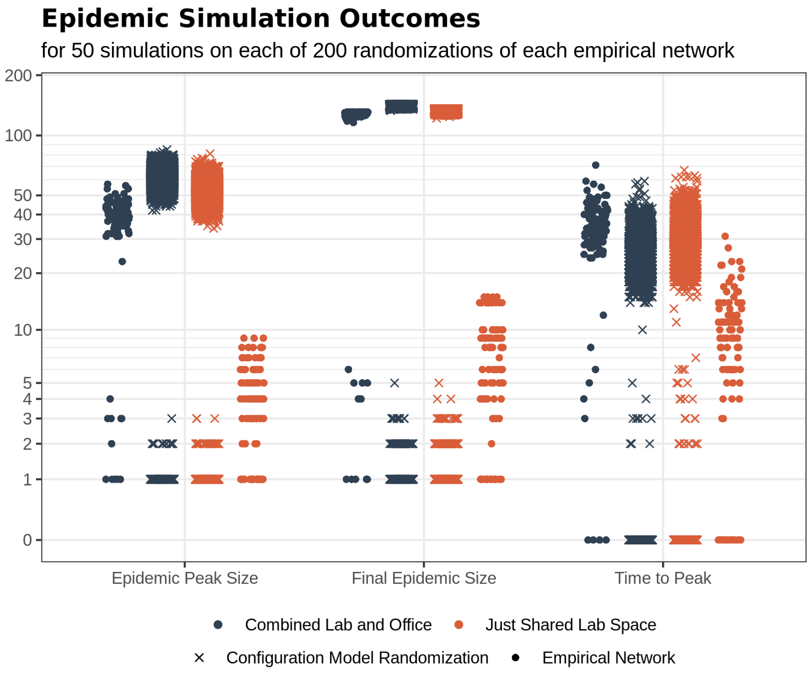

Figure S10: As fig. 7 in the main text, but including results from configuration model randomizations of the two empirical networks.

Isolated block model

As noted above, the configuration model does not accurately capture fragmented networks, instead producing networks with one large connected component and several very small components. This is particularly noticeable when comparing the random graphs to the shared lab-space network. To compensate for this, we ran additional simulations that generated a network of approximately the same size (*i.e.* number of individuals) and distribution of isolated cluster sizes as the network of shared lab-space. Note that we did not, however, recreate the few cases where two lab-spaces are linked by a single individual. Instead, in these simulated networks all individuals within a lab are connected to all other individuals in the same lab. Put another way, in contrast to the empirical network, in these simulated networks the shortest path between any two individuals (assuming such a path exists) is always one. We did this by generating a number of small, fully connected graphs, whose size was randomly drawn from the distribution of component sizes in the original lab-space network, and then merging all of these components into a single network for further analysis.

These synthetic networks better captured the structure of the shared lab-space network (fig. S11, and were able to recapitulate the disease dynamics seen on the empirical lab-space network (fig. S12).

Additional packages used in these simulations include `tidyverse` [Wickham et al., 2019], `tidygraph` [Pedersen, 2020], and `janitor` [Firke, 2020].

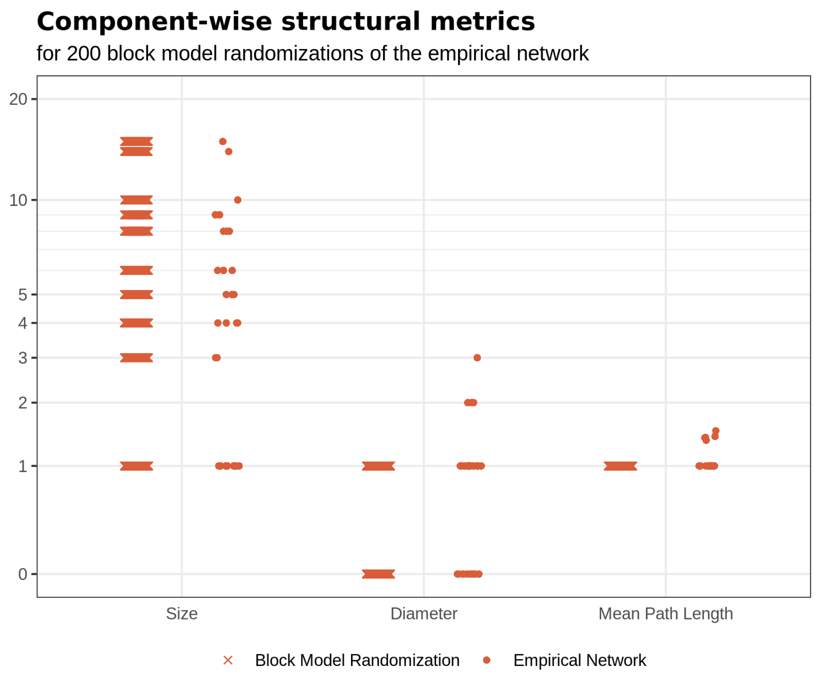

Figure S11: As fig. S7 in the supporting information and fig. S8 above, but including results from a block-model randomization of the two shared lab-space network.

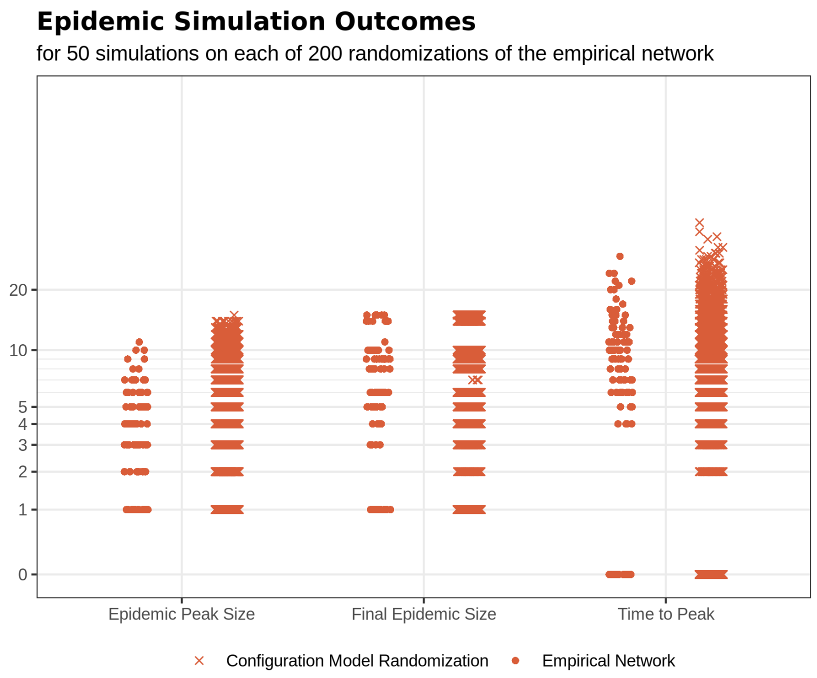

Figure S12: As fig. 7 in the main text and fig. S10 above, but including results from a block-model randomization of the shared lab-space empirical network.

References

Gabor Csardi and Tamas Nepusz. The igraph software package for complex network research. *InterJournal*, Complex Systems:1695, 2006. URL `http://igraph.org`.

Sam Firke. *janitor: Simple Tools for Examining and Cleaning Dirty Data*, 2020. URL `https://CRAN.R-project.org/package=janitor`. R package version 2.0.1.

Thomas Lin Pedersen. *tidygraph: A Tidy API for Graph Manipulation*, 2020. URL `https://CRAN.R-project.org/package=tidygraph`. R package version 1.2.0.

R Core Team. *R: A Language and Environment for Statistical Computing*. R Foundation for Statistical Computing, Vienna, Austria, 2020. URL `https://www.R-project.org/`.

Hadley Wickham, Mara Averick, Jennifer Bryan, Winston Chang, Lucy D'Agostino McGowan, Romain François, Garrett Grolemund, Alex Hayes, Lionel Henry, Jim Hester, Max Kuhn, Thomas Lin Pedersen, Evan Miller, Stephan Milton Bache, Kirill Müller, Jeroen Ooms, David Robinson, Dana Paige Seidel, Vitalie Spinu, Kohske Takahashi, Davis Vaughan, Claus Wilke, Kara Woo, and Hiroaki Yutani. Welcome to the tidyverse. *Journal of Open Source Software*, 4(43):1686, 2019. doi: 10.21105/joss.01686.
